# Real-time signal processing enabled by fused networks on a memristor-based system on a chip

**DOI:** 10.1126/sciadv.adv3436

**Published:** 2025-07-25

**Authors:** Zixu Wang, Wenhao Song, Tong Wang, Zihan Wang, Yichun Xu, Mingyi Rao, Fuxi Cai, Wenbo Yin, Mike Shuo-Wei Chen, Ning Ge, Maxwell Collins, Kangjun Bai, Sabyasachi Ganguli, Michael R. Page, Qing Wu, Linda Katehi, Qiangfei Xia, Miao Hu, J. Joshua Yang

**Affiliations:** ^1^ University of Southern California, Los Angeles, CA 90089, USA.; ^2^ TetraMem Inc., Fremont, CA 95131, USA.; ^3^ Information Directorate, Air Force Research Laboratory, Rome, NY 13441, USA.; ^4^ Materials and Manufacturing Directorate, Air Force Research Laboratory, Dayton, OH 45433, USA.; ^5^ Department of Electrical & Computer Engineering, Texas A&M University, College Station, TX 77843, USA.; ^6^ Department of Electrical & Computer Engineering, University of Massachusetts Amherst, Amherst, MA 01003, USA.

## Abstract

The von Neumann bottleneck has led to a substantial rise in energy consumption of computing hardware and memory systems, particularly for data-intensive tasks like signal processing. Memristor-based in-memory computing offers an efficient alternative by performing computations within analog memory. Here, we demonstrate real-time signal processing using a fused network that combines the real-time discrete Fourier transform (DFT) and convolutional neural network (CNN) on a memristor-based analog system on a chip (SoC). A 128-by-128 memristor crossbar array performs the DFT on audio signals with a peak signal-to-noise ratio of 33.49 dB, while the following CNN classifies the resulting spectrograms with 94.72% accuracy on the AudioMNIST dataset. In addition, convolution-based edge detection is applied to real-time video frames. The SoC offers substantial energy efficiency improvement over traditional digital systems in signal processing tasks. This work highlights the potential of memristor-based SoCs for efficient real-time signal processing.

## Teaser

A fused network of DFT and CNN is implemented on a memristor-based SoC to enable efficient real-time signal processing.

## INTRODUCTION

The rapid expansion of real-time signal processing applications, such as audio and video processing, has created a need for efficient and scalable signal analysis solutions. Traditional digital systems, while adaptable, face critical challenges in meeting the high-speed computational demands of these applications. The energy-intensive nature of analog-to-digital converters (ADCs) (*1*); substantial data movement requirements, i.e., the von Neumann bottleneck (*2*); and the inherent complexity of digital computations (*3*) all contribute to these limitations. These challenges become pronounced in environments requiring low latency and high energy efficiency, such as edge computing and Internet of Things devices. Achieving a balance between performance and energy efficiency has thus become a critical bottleneck for advancing next-generation signal processing systems.

Memristors are a leading memory candidate (*4*, *5*) that can perform in-memory computing, offering a more energy-efficient alternative to power-hungry digital signal processors while overcoming the von Neumann bottleneck. Unlike conventional designs, where memory and computation are separate, memristors allow data to be processed directly within the memory (usually the memristor crossbar array), reducing the need for energy-intensive and time-consuming data transfers (*6*–*10*). One of their key strengths is the ability to perform vector-matrix multiplication (VMM), a basic operation in many signal-processing and machine learning (ML) applications, in a single step (*11*–*13*). This capability not only speeds up computation but also lowers latency, making memristors a great fit for tasks that need real-time performance. These qualities make memristor-based systems especially promising for real-time multimedia applications, where high speed and low power usage are crucial.

Many hardware systems have been developed that leverage memristor crossbar arrays to perform VMM, improving accuracy and energy efficiency. Examples include memristor-based neural network accelerators designed to manage computationally intensive operations like matrix multiplications used in neural networks (*14*–*20*). There are also hardware-implemented neural networks, such as the convolutional neural network (CNN) (*21*), long short-term memory networks (*22*, *23*), and spiking neural networks (*24*–*29*), which demonstrate how memristors can support the implementation of complex ML models in hardware. To further enhance the accuracy of VMM operations, researchers are improving memristor materials and refining circuit designs to minimize issues such as nonlinearity and variability (*30*–*33*). Advanced training and calibration techniques and the hybrid designs combining memristors with complementary metal-oxide semiconductor technology have also been explored to improve precision and reliability (*12*, *34*–*41*).

Beyond ML, memristor crossbars are being applied to a range of signal processing tasks. For instance, they have been used to perform discrete Fourier transform (DFT) (*42*, *43*), analog filtering (*44*–*46*), neural signal analysis (*47*–*50*), wireless communication (*51*), and audio or image processing directly within the array (*52*–*55*). However, notable challenges persist in current implementations: (i) Many of these signal processing applications rely on small-scale arrays or software simulations rather than large-scale, practical hardware systems; (ii) existing works lack real-time processing capabilities; (iii) despite numerous demonstrations, no memristor-based signal processing system has been fully integrated into a practical system on a chip (SoC); and (iv) signal processing and neural network have not been fused together as one network and demonstrated on a single chip. Overcoming these limitations will be essential for demonstrating and unlocking the full potential of memristor crossbars in signal processing.

To overcome these barriers, in this work, we proposed the first demonstration of a fused network implemented on a custom memristor-based SoC for real-time signal analysis. The concept of a fused network, which seamlessly combines both signal processing and ML together, allows one to efficiently run on hardware that is typically designed for general neural network models. We show that a 128-by-128 DFT matrix mapped on the memristor crossbar array can generate a spectrum for input audio signals, achieving a peak signal-to-noise ratio (PSNR) of 33.49 dB compared to error-free software-based results. Then, we present real-time convolution-based edge detection for video frame analysis, testing the versatility of the SoC in handling diverse signal processing workloads. Last, by fusing the DFT signal processing matrix into a CNN neural network, we proposed and demonstrated a fused network on SoC to directly and efficiently perform audio signal transformation and spectrum classification. The spectrograms from DFT are analyzed using a CNN, achieving a classification accuracy of 94.72% on the AudioMNIST dataset (see Materials and Methods, fig. S10, and Supplementary Note 3). The memristor-based SoC also achieves ~49× improvement in energy efficiency over a graphics processing unit (GPU; A100, 7 nm) when the SoC is scaled to 12-nm technology node. By combining DFT and convolutional operations on a compact, scalable SoC, this work highlights the potential of such systems in advancing energy-efficient, high-performance solutions for real-time multimedia and signal processing tasks.

## RESULTS

### DFT and convolution operation with VMM on a memristor crossbar array

**Fig. 1.**
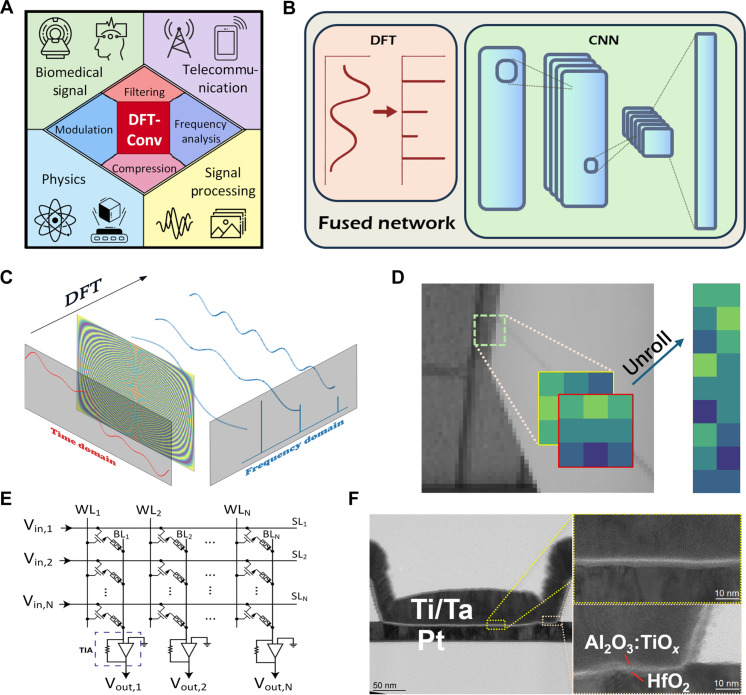
Signal processing using DFT and convolution kernels with the memristor crossbar array. (**A**) Applications of DFT and convolution across various domains, including biomedical signal processing, telecommunications, physics, and general signal processing. DFT enables tasks such as filtering, modulation, frequency analysis, and compression in these areas. (**B**) The fused network integrates the DFT and CNN module. The whole network is implemented by the SoC. (**C**) Transformation of a time-domain signal into the frequency domain using a DFT matrix with a VMM operation. In this example, the input signal 0.5sin(*t*) + 0.3sin(3*t*) + 0.2sin(5*t*) produces three distinct peaks in the frequency domain. (**D**) Convolution operation between a grayscale image and two kernels, where the kernels are unrolled into columns for compatibility with VMM operations. (**E**) Circuit design of the 1T1R array on the SoC. Input voltages are applied to SLs to control current flow from WLs to BLs, with accumulated currents converted to voltage signals for ADC readout. (**F**) Transmission electron microscopy image of a memristor in the 1T1R array. It has a Ti/Ta top electrode and a Pt bottom electrode. Scale bars, 50 nm (left) and 10 nm (right insets). The details of fabrication are in Materials and Methods.

The DFT and convolution operation are critical tools across a wide range of real-world applications, as illustrated in Fig. 1A. They play an important role in biomedical signal processing, aiding in the analysis of brain wave signals and medical imaging. In telecommunications, they enable tasks such as frequency analysis and signal filtering, which are essential for wireless communication and data compression. They also find applications in physics, where they are used to study wave phenomena and material properties, as well as in general signal processing tasks like convolution, filtering, and image processing. These diverse applications highlight the importance of efficient and scalable methods for performing DFT and convolution in modern computational systems. The ability to perform DFT and convolution efficiently is critical for real-time processing.

Figure 1B illustrates the architecture of a fused network that integrates a DFT operation with a CNN on a SoC. In this design, the DFT is implemented as the first layer of the network, allowing the transformation from the time or spatial domain to the frequency domain to be performed directly within the network. The subsequent CNN layers work directly with the frequency-domain representation for further processing. This seamless integration eliminates the need for separate data conversion, storage, and transfer between the DFT and CNN components, thereby reducing latency and improving computational efficiency. By tightly integrating preprocessing with deep learning, this architecture enables faster and more efficient performance in real-time applications.

Figure 1C demonstrates how DFT can be realized through VMM operations. In this example, a composite signal x (t)=0.5sin (t)+0.3sin (3t)+0.2sin (5t) is transformed from the time domain to the frequency domain using VMM between the input waveform and the DFT matrix, where the input waveform is sampled and projected onto the DFT matrix, producing its frequency components. This method demonstrates the effectiveness of VMM in performing the DFT. Figure 1D illustrates how convolution can be implemented using VMM operations. The core idea is to unroll every convolution kernel into a single column and arrange them into a matrix, where each row corresponds to a flattened version of a kernel. The input signals are similarly flattened and multiplied by this kernel matrix using a VMM operation. This approach enables the convolution process to be executed in parallel, increasing efficiency compared to traditional, sequential methods.

Figure 1E presents the architecture of a one-transistor-one-resistor (1T1R) array, a configuration designed to perform VMM operations. In this structure, each cell consists of a transistor (T) and a resistive element (R), with the transistors controlling access to the resistive cells. The word lines (WLs) and bit lines (BLs) form the crossbar array, enabling selective activation of rows and columns for computation. Input voltages $V_{\text{in}}$ are applied to the select lines (SLs), while the resulting currents, weighted by the resistive states of the cells, are summed along the BLs. These currents are then converted to output voltages $V_{\text{out}}$ through transimpedance amplifiers (TIAs), completing the VMM operation in a highly parallel manner. Figure 1F shows a high-resolution transmission electron microscopy image of a memristor within the 1T1R array, providing a detailed cross-sectional view of its nanoscale structure. The memristor consists of a Pt bottom electrode and a Ti/Ta top electrode, with a resistive switching layer (HfO_2_/Al_2_O_3_). The details of the fabrication of the 1T1R array are provided in Materials and Methods. Figure S1 shows the normal switching behaviors of the memristor device. This nanoscale design enables high density and scalability, which are crucial for implementing efficient VMM operations in modern analog computing systems. Together, these figures highlight the practical realization of VMM hardware.

### Real-time audio processing with the DFT on a SoC

**Fig. 2.**
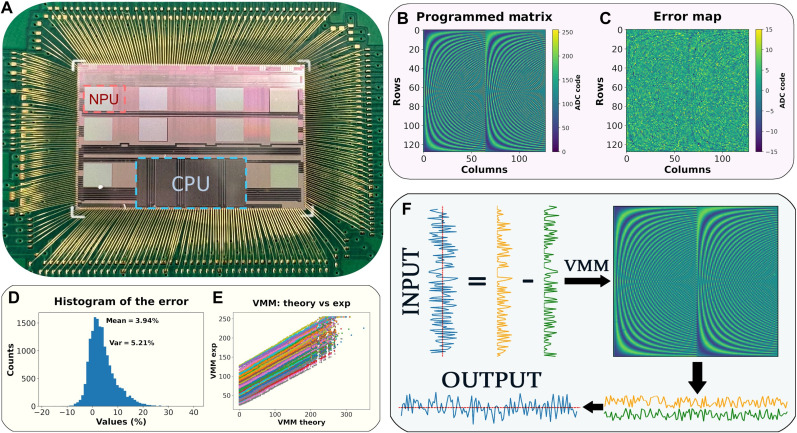
Performance analysis of the memristor-based SoC for DFT matrix programming and VMM. (**A**) Photograph of the unpackaged SoC, featuring 10 NPU cores (for example, the red rectangle in the dashed line represents one NPU), RISC-V CPU (blue rectangle), and other peripheral circuits, wire bonded to a PCB testing platform. (**B**) Measured programmed DFT matrix on the chip, with elements represented as 8-bit unsigned integers corresponding to ADC codes read from the chip. (**C**) Error map of the programmed DFT matrix. (**D**) Histogram of the programming error, showing a mean error of 3.94% and a variance of 5.21%. (**E**) Comparison of experimental VMM results with theoretical VMM results. Each color represents data from a specific ADC column in the memristor-based SoC. Data points for each ADC align closely along a straight line, indicating strong linearity of the SoC. (**F**) To accommodate the 8-bit unsigned integer input constraint of the DAC, the input signal is split into positive and negative components. VMM operations are performed separately for these components, and the results are subtracted to produce the final VMM output.

Figure 2A presents a photograph of the unpackaged circuit, which includes 10 neural processing unit (NPU) cores and peripheral circuits wire bonded to a printed circuit board (PCB) testing platform. Each NPU core contains a 256-by-256 memristor crossbar array and the surrounding peripheral circuitry, including a digital-to-analog converter (DAC), ADC, TIA, etc. The photo of the SoC is shown in fig. S2. The detailed information on the SoC chip is provided in Materials and Methods and the Supplementary Materials (see figs. S3 and S4).

The performance of programming the memristors on the SoC is evaluated using a customized DFT matrix, as shown in Fig. 2B. The details of the matrix generation are provided in Materials and Methods and fig. S6. Figure 2C presents the error map of the programmed matrix, illustrating that the deviations are uniform and fall within a narrow range across the matrix elements. The programmed matrix on the chip closely matches the target DFT matrix, achieving a root-mean-square error of 6.40%, which demonstrates the high fidelity of the memristor programming process.

The error distribution is further analyzed in Fig. 2D and fig. S7, which shows a histogram of the programming errors. The distribution has a mean of 3.94% and a variance of 5.21%, indicating that the errors are centered near zero with a narrow spread. This low error rate ensures that the memristor crossbars can reliably store and process complex matrices like the DFT matrix.

The input data should be first sampled and quantized to an 8-bit integer and then transferred to the SoC using the cable with the Serial Peripheral Interface (SPI) protocol (see fig. S3). The throughput of the SoC is measured and shown in fig. S8. To handle input signals containing both positive and negative values, the SoC uses a process that ensures accurate VMM operations. As shown in Fig. 2F, the input signal is first split into its positive and negative components. Each component is processed separately in two VMM operations using the same memristor crossbar arrays. Once both computations are complete, the results are subtracted to produce the final VMM result. The details are provided in Supplementary Note 1.

The results of the VMM operation are presented in Fig. 2E, where the experimental VMM outputs are plotted against the theoretical VMM results from 500 sample inputs. Each color represents the results obtained from a specific ADC column in the memristor-based SoC. The data points for each ADC align closely along a straight line, demonstrating the high linearity of the VMM calculations performed by the system. The correlation coefficients, which are all greater than 0.995 (see fig. S5), confirm the strong agreement between experimental and theoretical results. However, each line has a distinct intercept and slope, which are attributed to fixed offset values inherent to individual ADCs because of manufacturing variations and the IRdrop issue in the array. These offsets and slope differences, although consistent across operations, introduce slight deviations in the output. Fortunately, such nonidealities can be effectively corrected through postprocessing once the results are obtained (see Supplementary Note 2 for details). This high level of linearity, combined with the ability to address ADC-specific variations, underscores the reliability and accuracy of the SoC for performing large-scale VMM operations.

### Real-time video processing with convolution on the SoC

**Fig. 3.**
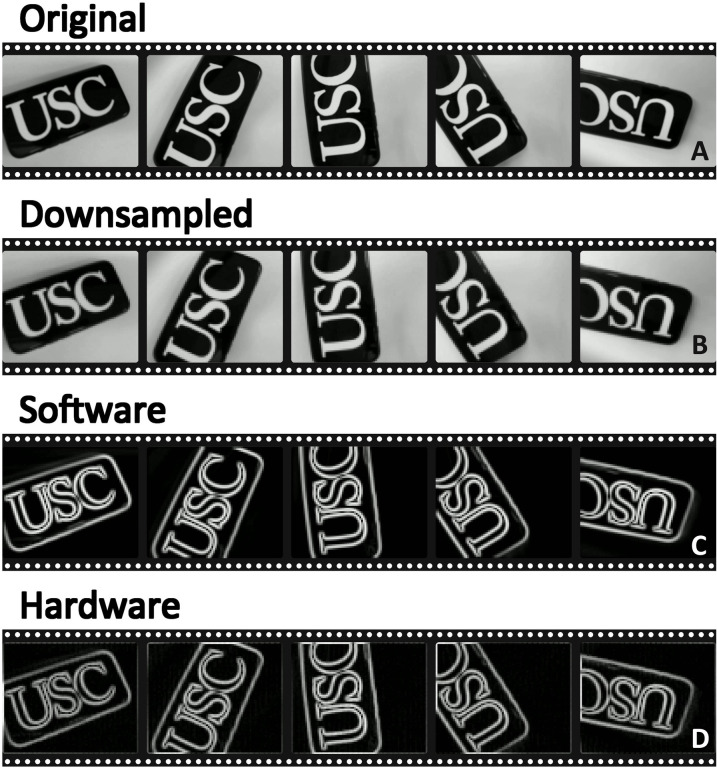
Real-time edge detection of video streams using convolution implemented on the SoC. The figure demonstrates a real-time video processing example where the integrated SoC performs edge detection on the real-time video captured by the camera. The figure shows five frames in the video, with a time interval of 1 s. (**A**) Original video frames captured by the camera, depicting the letter “USC” in its full resolution. The video has a frame rate of 10 fps. (**B**) Downsampled version of the frames. It is resized to a resolution of 64 by 78 pixels to fit the SoC’s input requirements. (**C**) Result of software-based edge detection applied to the downsampled frames, indicating a clear and detailed edge of the letters on the screen. (**D**) Output of the SoC’s hardware-based edge detection, which also identifies edges in the scene. This hardware-processed output achieves a PSNR of 30.43 dB, demonstrating the SoC’s capability to do convolution and perform edge detection efficiently in real time.

In the real-time video processing demonstration, the integrated SoC is used to perform edge detection using a convolution operation with the Sobel kernel (see Materials and Methods). The video stream is captured by a camera. Figure 3 shows an example of video frames processed in real time through the system. Figure 3A represents the original frames, depicting the letters “USC” on a screen as captured by the camera. Figure 3B is the downsampled version of the original frames, reduced to a resolution of 64 by 78 pixels to match the input size of the processing pipeline. Figure 3C shows the result of software-based edge detection applied to the downsampled frames. This output highlights the outlines of letters in the scene, providing a clear and well-defined edge map. In contrast, Fig. 3D is the result of hardware processing on the SoC, which also extracts edges from the frames. We can see the intensified edge of the image clearly. The hardware-processed output achieves a PSNR of 30.43 dB compared to the software result (see Materials and Methods for the calculation of PSNR and Supplementary Note 4 for error analysis), demonstrating the capability of the SoC to perform real-time edge detection. The complete video is available in movie S1.

### Real-time processing and classification with DFT + CNN fused network on the SoC

By combining the DFT matrix and CNN, we use a hardware-implemented fused network to perform real-time classification of audio signals. Spoken digit recognition is used as an example for demonstration here because it is a fundamental task in speech processing, which has wide-ranging applications, including voice-controlled systems, automated customer service, and speech-to-text technologies.

**Fig. 4.**
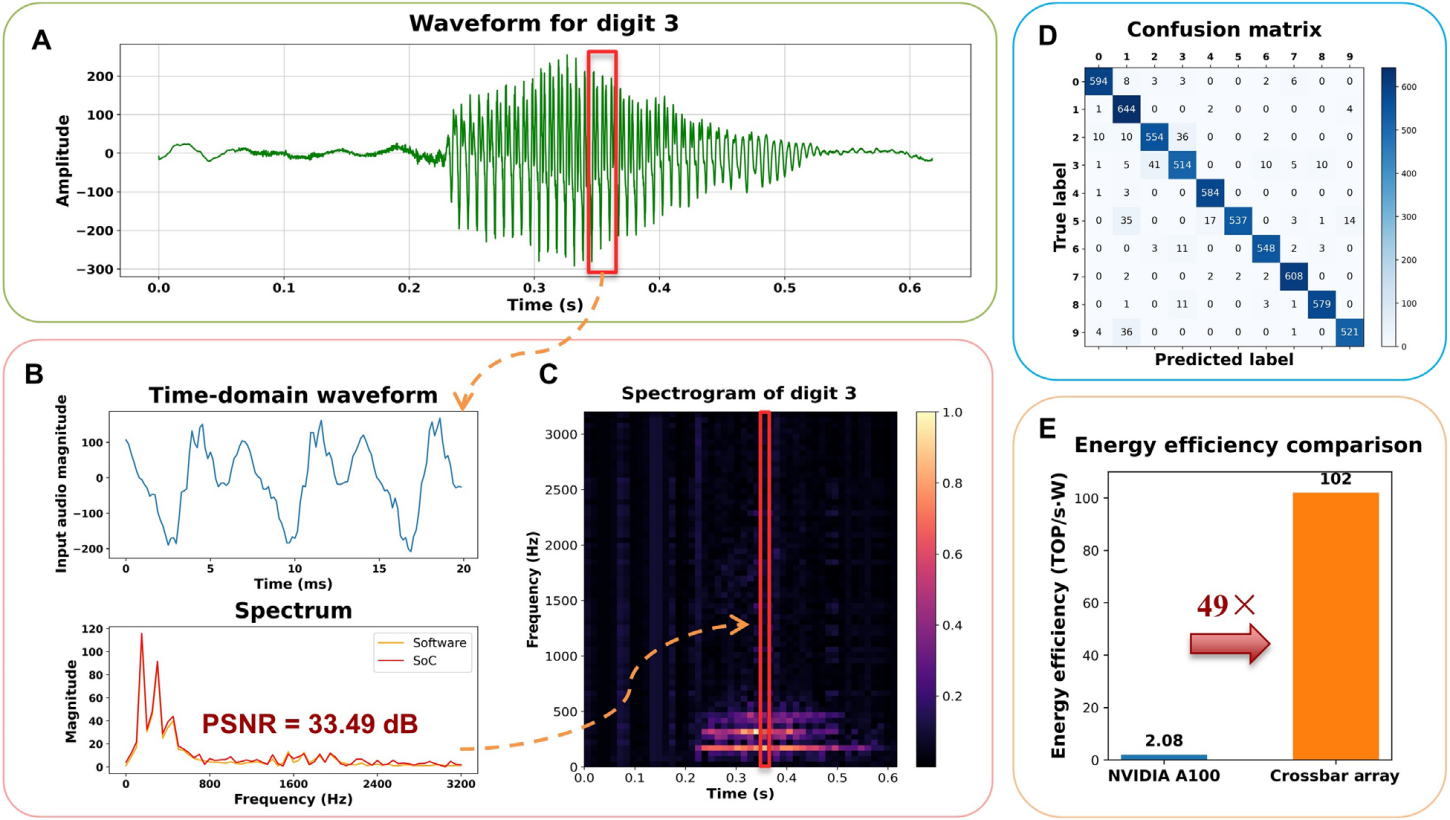
Real-time recognition of human-spoken digits using the fused network on the SoC. (**A**) Recorded waveform from the microphone representing the spoken digit “3.” The input signal is sliced into 20-ms segments with a 10-ms stride to better capture local features. (**B**) Spectrum of a single slice calculated by software (red) and hardware (yellow), demonstrating a PSNR of 33.49 dB. (**C**) Spectrogram constructed by arranging the spectra of all slices, with time on the *x* axis and frequency on the *y* axis. The spectrogram preserves all essential waveform information for CNN-based classification. (**D**) Confusion matrix showing recognition probabilities for each digit. The DFT + CNN implemented on the SoC achieves a classification accuracy of 94.72%. (**E**) Energy efficiency comparison: The core crossbar array achieves 102 TOP/(s·W) when scaled to 12 nm, outperforming the NVIDIA A100 GPU by a factor of 49 in VMM operations.

Figure 4A illustrates the waveform of the spoken digit “3,” displaying its amplitude over time, which is normalized to −255 to 255. The waveform spans ~0.6 s, with distinct peaks corresponding to the vocalized energy of the digit.

To process the spoken digit, first, the input voice signal is divided into multiple slices. These slices capture the local time-domain features of the waveform, breaking them into smaller, manageable segments for DFT analysis. The upper part of Fig. 4B is an example of one of the slices. The lower part of Fig. 4B displays the calculated spectrum of this slice, computed using the customized DFT matrix shown in Fig. 2B (a video of real-time spectrum calculation is attached in movie S2). The results from the hardware closely align with those calculated by the software, achieving a PSNR of 33.49 dB. This close agreement highlights the accuracy of the SoC in performing DFT operations.

Once the spectra for all slices are computed, they are arranged as columns to construct a spectrogram, as shown in Fig. 4C for the spoken digit “3.” The spectrogram provides a detailed time-frequency representation of the signal, with peaks in the spectrum corresponding to bright spots in the spectrogram, such as those bright spots in the red-marked region. This image representation of audio signal captures the essential features of the spoken digit and can serve as input to a neural network for classification. Using the TetraMem Instinct 1.4 software development kit described in Materials and Methods, we quantized and implemented the classifier on the SoC using DFT as its first layer and CNN as its next four layers (see Supplementary Note 3 for details). When evaluated on the AudioMNIST dataset, this classifier achieves a high classification accuracy of 94.72%. Figure 4D presents the confusion matrix for the test dataset, illustrating the performance across all 10 classes. The integration of hardware-implemented DFT and CNN enables real-time spoken digit recognition, demonstrating both speed and accuracy. A video showing the real-time classification process is included in movie S3.

Figure 4E highlights the energy efficiency comparison between the NVIDIA A100 GPU and the memristor crossbar array implemented on our SoC when scaled to 12 nm. The crossbar array achieves an energy efficiency of 102 tera-operations (TOPs)/W, vastly outperforming the 2.08 TOPs/W of the NVIDIA A100 GPU. This demonstrates an ~49× improvement in energy efficiency, underscoring the potential of our SoC for high-performance, low-power applications, particularly in real-time audio processing and ML tasks.

## DISCUSSION

This work demonstrates the potential of memristor-based analog SoCs for real-time signal processing tasks, including spectrum calculation, video edge detection, and spoken digit recognition. By integrating hardware-implemented DFT and convolution operations with a CNN classifier, the system achieves high accuracy and efficiency in analyzing real-time signals. The use of memristor crossbar arrays enables highly parallel VMM operations, offering a notable energy efficiency improvement compared to state-of-the-art GPUs. These advancements showcase the feasibility of using memristor-based systems for low-power, high-performance applications.

However, some limitations and areas for improvement must be addressed to fully realize the practical applications of this technology. A major limitation lies in the design of the data conversion pipeline. In principle, the tasks demonstrated in this study do not strictly require analog-to-digital and digital-to-analog conversions. A more energy-efficient system could bypass the digitization stage altogether by directly connecting the analog input sources to the memristor crossbar arrays through analog front-end circuits. Similarly, the analog output from the DFT matrix could be directly fed into the CNN without digitization, eliminating the need for intermediate analog-to-digital and digital-to-analog conversions. Such an approach could substantially reduce energy consumption while simplifying the system architecture.

Moreover, expanding the size of the memristor crossbar arrays could unlock additional capabilities. Larger arrays would allow for a finer resolution of the spectrum, enabling more detailed analysis of signals. For video processing, this could facilitate handling larger frame sizes, improving the quality and applicability of the tasks. Such advancements would result in a more powerful accelerator capable of supporting a broader range of applications with enhanced precision and scalability.

Last, the speed bottleneck of VMM operations on the current SoC is not in the analog domain but rather in the digital circuits managing data flow and processing. The digital components of the SoC are not yet optimized for high-speed operations, limiting the overall throughput. Future iterations of the SoC could address this limitation by incorporating faster and more powerful digital circuits, enabling larger and faster VMM operations. These advancements would further enhance the performance and scalability of the system, bringing it closer to practical deployment in a wide range of applications.

## MATERIALS AND METHODS

### 1T1R array fabrication

The 1T1R arrays were fabricated by integrating transistors and memristors on a single platform using a commercial 180-nm process line. Transistors were first structured with exposed tungsten vias, followed by surface oxide cleaning to prepare for memristor integration. Pt bottom electrodes were sputtered and lithographically patterned onto the vias. A 100-nm SiO_2_ isolation layer was subsequently deposited and etched to create cavities terminating at the Pt surface.

Memristor devices were formed within these cavities by sequentially depositing a bilayer HfO_2_/Al_2_O_3_ resistive switching layer via atomic layer deposition and a Ti/Ta top electrode via sputtering. Last, aluminum interconnects were patterned to route the top electrodes to bond pads, enabling electrical testing.

### MX100 SoC evaluation kit

We used the TetraMem MX100 SoC evaluation kit, a combination of hardware (MX100 SoC) and software stack (Instinct 1.4), to demonstrate our signal processing system using analog in-memory computing. The hardware platform includes a memristive SoC mounted on a customized PCB alongside essential peripherals housed in integrated circuits. The SoC features 10 memristive computing cores, a RISC-V central processing unit (CPU), a data bus, and digital peripherals. Each memristive computing core consists of a 256-by-256 1T1R crossbar array (with 8-bit memristors) and peripheral circuits, including TIAs, DACs, ADCs, and control circuits. A programming circuit enables the cores to alternate sequentially between programming and computing modes.

In addition, digital circuits within the cores implement general functions such as scaling ADC outputs, compensating for column-wise analog gain and offset errors, and neural network operations, including normalization and activation functions like ReLU and Sigmoid. The SoC also includes a two-stage RISC-V CPU based on the PULPino platform; a high-bandwidth Advanced eXtensible Interface bus for connecting the cores, CPU, and peripherals; and a scatter-gather direct memory access engine for efficient data transfer.

The software platform, TetraMem Instinct 1.4 software development kit, comprises three major components: the ML model quantizer, ML compiler, and deployment tool. The ML model quantizer transforms standard ML inference models to operate with 8-bit unsigned integer tensors and operators. The ML compiler allocates memory space for tensors and buffers within the SoC, maps selected operators onto dedicated hardware, partitions large operators for parallel execution, and schedules parallel tasks across the SoC’s on-chip facilities. Meanwhile, the deployment tool provides components such as a cross-compiler, ML runtime, and VMM calibration tools, ensuring the seamless deployment of compiled operations and models onto the SoC.

For this demonstration, we used the ML compiler and deployment tool to quantize and implement analog VMM for the DFT calculation. Furthermore, the ML model quantizer was used to quantize the pretrained CNN, which was implemented for spoken digit classification using the ML compiler and deployment tool. These capabilities highlight the MX100 SoC’s potential for energy-efficient and high-performance audio signal processing.

### DFT matrix

The DFT matrix is an *N* × *N* complex matrix (*N* = 128 here), where each element in the *j*th row and *k*th column is defined as

$$W_{\mathrm{jk}}=e^{-\frac{2\pi \;\mathrm{ijk}}{N}}$$ (1)

Here, *j* and *k* are integers ranging from 0 to *N* − 1.

For a given input vector $x=\left[x_{0},x_{1},\ldots ,x_{N}\right]$ , the Fourier transform of x , denoted as X, can be expressed as the VMM

X=Wx (2)

However, the memristor crossbar array is designed to store scalar weights and cannot directly accommodate the complex weights of the DFT matrix. Therefore, a mapping method is required to adapt the original complex matrix to the array.

It is important to notice that the elements of the DFT matrix exhibit conjugate symmetry

$$W_{\mathrm{jk}}=W_{\left(N-j\right)k}^{*}$$ (3)

In addition, the dc component and the Nyquist frequency component, corresponding to the 0th and *N*/2-th columns of the DFT matrix, are purely real (their imaginary parts are zero). As a result, the information from the original complex DFT matrix can be fully represented by the following components:

1) Real parts of the 0th to *N*/2-th columns.

2) Imaginary parts of the 1st to (*N*/2 − 1)-th columns.

This reduced representation preserves all essential information while substantially minimizing storage requirements. However, the range of this customized DFT matrix is [−1, 1]; we need to map the weights to be 8-bit unsigned integers to correctly program the matrix on the SoC. The method is to get the target matrix by the following equation

$$G_{\text{target}}=G_{\text{DFT}}\cdot D_{s}+b$$ (4)

where $G_{\text{DFT}}$ is the customized DFT matrix, $D_{s}$ is a diagonal matrix of scaling parameters, and b is an offset value to shift all the elements to the positive region, because memristor’s conductance cannot be negative.

Following the VMM operation on the target DFT matrix on the SoC, the magnitude of the spectrum is obtained by calculating the square root of the sum of the squared values of both the real and imaginary components by a computer. A detailed visualization of this customization is provided in fig. S6.

### Audio digit recognition task

The audio sample shown in Fig. 4 is taken from the AudioMNIST dataset (*56*), which contains recordings of human-spoken digits ranging from 0 to 9. The details on how we use this dataset are provided in Supplementary Note 3. For the real-time demonstration, a microphone with a sampling rate of 6.4 kHz is used to capture the input signal. The captured signal is scaled to the range of [−255,255] and then divided into overlapping slices, each 20 ms long with a stride of 15 ms. This slicing process ensures that the local time-domain features of the waveform are preserved, enabling effective analysis.

The CNN used for classification consists of four convolutional layers, designed to process the spectrograms generated from the input audio slices. The quantized accuracy of the system, reflecting the classification performance of CNN after adapting it to hardware constraints, matches closely with its baseline accuracy, demonstrating minimal loss in precision. The hardware-implemented system also achieves high accuracy, validating the effectiveness of the memristor-based SoC in real-time spoken digit recognition. Detailed performance metrics, including quantization and hardware accuracy, are discussed in Supplementary Note 3.

### Edge-detection of real-time video

In real-time video processing, a video is treated as a sequence of continuous frames, allowing edge detection to be applied to each frame individually. The Sobel kernel is a commonly used filter in image processing for detecting edges. It acts as a discrete differentiation operator, emphasizing regions of high spatial gradients that correspond to edges in an image. The Sobel operator calculates gradients in two perpendicular directions ( $G_{x}$ and $G_{y}$ ) using the following kernels

$$\begin{array}{c} G_{x}=\left[\begin{array}{ccc} -1 & 0 & 1\\ -2 & 0 & 2\\ -1 & 0 & 1 \end{array}\right]\cdot A,\;G_{y}=\left[\begin{array}{ccc} -1 & -2 & -1\\ 0 & 0 & 0\\ 1 & 2 & 1 \end{array}\right]\cdot A \end{array}$$ (5)

where *A* represents the input frame.

The overall edge strength of the frame is then calculated as the magnitude of the gradients, approximated by

$$G\approx |G_{x}|+|G_{y}|$$ (6)

### Calculation of the PSNR value

The PSNR quantifies the fidelity between an original signal x and its reconstructed or degraded version $\hat{x}$ . To compute the PSNR, first calculate the mean squared error (MSE) by averaging the squared differences between corresponding samples of the two signals

$$\text{MSE}=\frac{1}{N}\sum_{i=1}^{N}\left(x_{i}-\hat{x_{i}}\right)$$ (7)

where *N* is the total number of samples. The PSNR is then derived using the ratio of the maximum signal amplitude to the MSE

$$\text{PSNR}=10\cdot {\text{log}}_{10}\left(\frac{{\text{MAX}}_{x}^{2}}{\text{MSE}}\right)$$ (8)

Higher PSNR values indicate better signal quality. This metric is widely applied in image, video, and audio processing to evaluate compression or enhancement algorithms.

## Supplementary Materials

### The PDF file includes:

Supplementary Notes S1 to S4 Figs. S1 to S12 Legends for movies S1 to S3
