# Supplementary material for: Real-time signal processing enabled by fused networks on a memristor-based system on a chip
Source: Sci Adv. 2025 Jul 25;11(30):eadv3436. doi: 10.1126/sciadv.adv3436 (PMC12292901; doi:10.1126/sciadv.adv3436)
Supplement: Supplementary file 1 — Supplementary Notes S1 to S4 Figs. S1 to S12 Legends for movies S1 to S3 [file sciadv.adv3436_sm.pdf]

Science Advances
AAAS

Supplementary Materials for

Real-time signal processing enabled by fused networks on a memristor-based system on a chip

Zixu Wang *et al.*

Corresponding author: J. Joshua Yang, jjoshuay@usc.edu; Miao Hu, miao.hu@tetramem.com

Sci. Adv. **11**, eadv3436 (2025)
DOI: 10.1126/sciadv.adv3436

The PDF file includes:

Supplementary Notes S1 to S4
Figs. S1 to S12
Legends for movies S1 to S3

Other Supplementary Material for this manuscript includes the following:

Movies S1 to S3

Supplementary Note 1: Vector-Matrix Multiplication (VMM) operation with positive voltages

Due to the limitations of the on-chip digital-to-analog converters (DACs), the input voltages applied to the memristor crossbar array must remain in the positive range. To accommodate VMM with input vectors containing negative values, the input vector V_{in} is split into two components: V_{in}^{+} and V_{in}^{-}. These components are generated through element-wise comparison as follows:

$$V_{in}^{+}(i) = \max(V_{in}(i), 0)\,, \qquad V_{in}^{-}(i) = -\min(V_{in}(i), 0) \qquad (S1)$$

The relationship between these components and the original input vector is expressed as:

$$V_{in} = V_{in}^{+} - V_{in}^{-} \qquad (S2)$$

To compute the output current signal, two separate VMM operations are performed using the programmed conductance matrix G_{array}:

$$I_{out}^{+} = V_{in}^{+} \cdot G_{array}, \qquad I_{out}^{-} = V_{in}^{-} \cdot G_{array} \qquad (S3)$$

The final output current is then obtained by subtracting the results of these two operations:

$$I_{out} = I_{out}^{+} - I_{out}^{-} = (V_{in}^{+} - V_{in}^{-}) \cdot G_{array} = V_{in} \cdot G_{array} \qquad (S4)$$

This method not only resolves the issue of DACs being unable to generate negative voltage signals but also sparsifies the input vectors. This sparsity enables the analog-to-digital converter (ADC) to utilize a larger gain parameter, fully utilizing the sensing range and thereby enhancing the accuracy of the results.

Supplementary Note 2: Calibration of VMM results

Ideally, all ADCs on the system-on-chip (SoC) should exhibit identical behavior. However, due to manufacturing variations and differing operating conditions, ADCs may exhibit non-idealities such as non-linearity, gain and offset errors, and noise. As shown in **Fig. 2E**, the ADCs demonstrate good linearity, but there are slight variations in gain and offset values. Consequently, calibration is required to ensure the raw ADC outputs from the SoC align with the expected results.

The non-ideal behavior of the ADCs is modeled as:

$$V_{theo} = a \cdot V_{exp} + b \qquad (S5)$$

where V_{theo} and V_{exp} are the theoretical and experimental results, respectively, as shown in Figure 2e. The parameters a and b represent the gain and offset coefficients obtained through linear fitting of the experimental data.

After acquiring the raw output V_{raw} from the SoC, the calibrated result $V_{calibrated}$ is calculated using the following equation:

$$V_{calibrated} = a \cdot V_{raw} + b \qquad (S6)$$

This calibration method enhances the reliability and precision of the analog VMM results, effectively mitigating systematic errors caused by ADC variations. By applying this approach, the results are better aligned with theoretical expectations, ensuring accurate and consistent performance across the SoC.

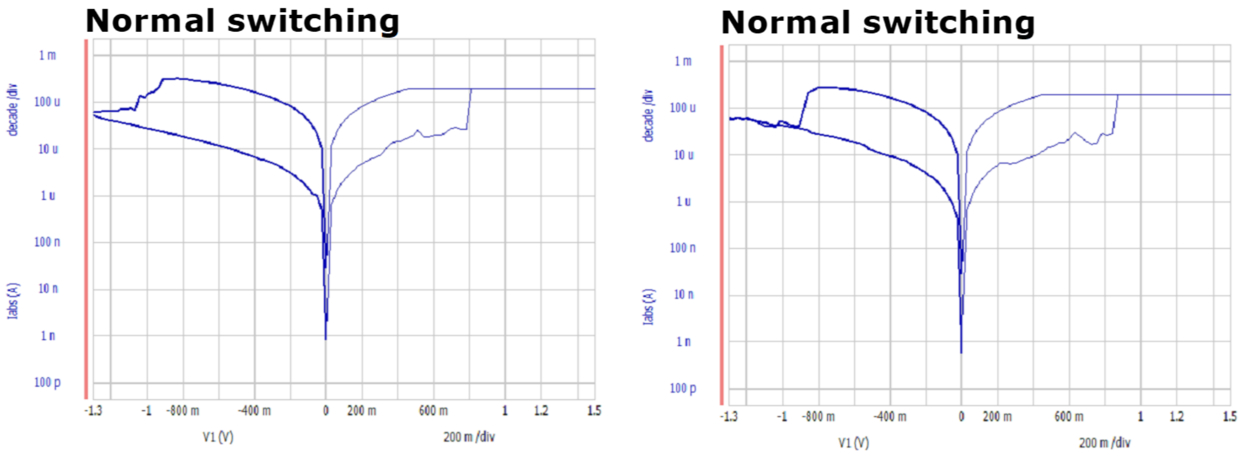

Fig. S1. Normal switching behavior of two memristor devices. This figure shows the set and reset behavior measured by Keysight B1500A semiconductor device analyzer of two memristors, which are the same devices as that on our SoC.

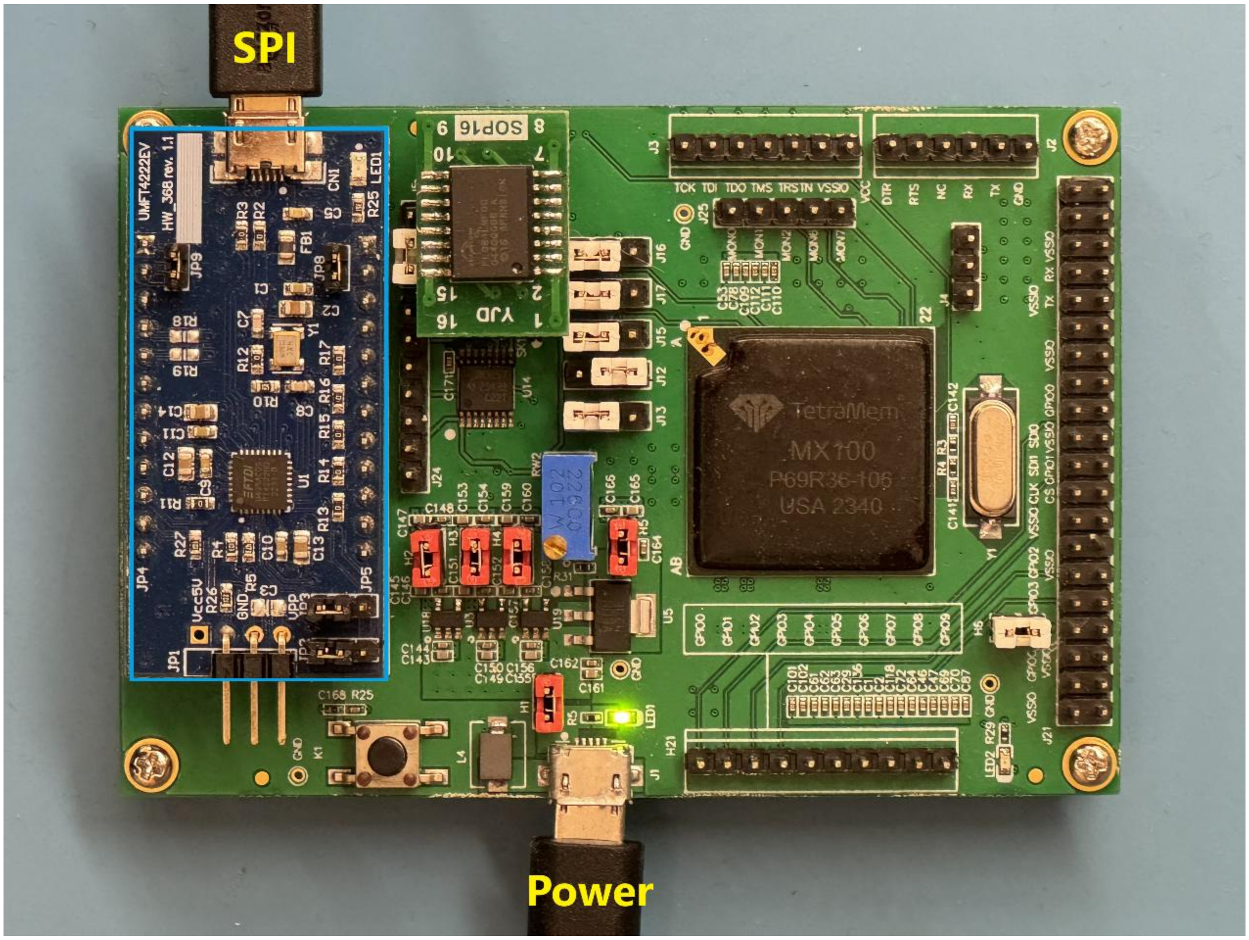

Fig. S2. Photograph of the SoC. The chip connects to the host machine via the FT4222 chip, which is enclosed within a blue frame in the image, using the SPI communication protocol. Power is supplied through a Micro USB cable, providing a compact and efficient setup.

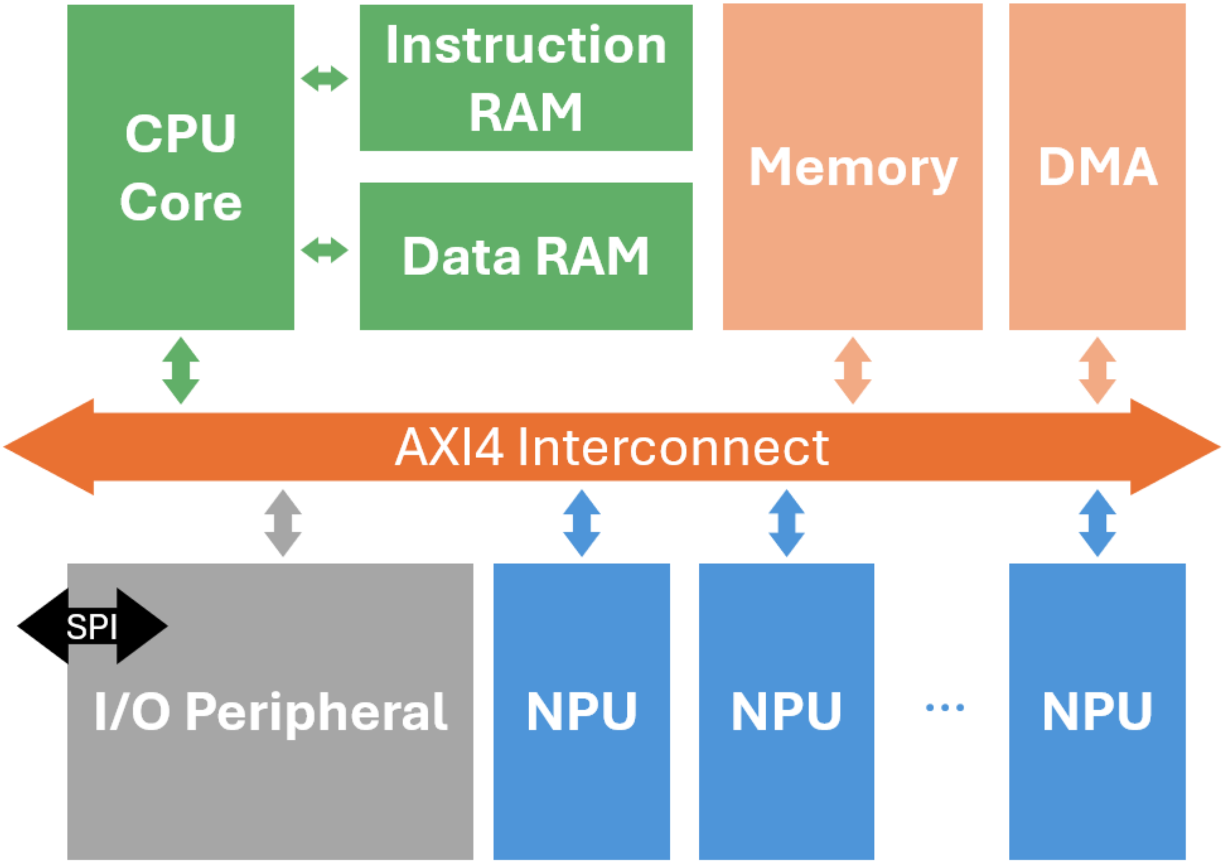

Fig. S3. Architecture of SoC. The SoC integrates ten neural processing unit (NPU) cores, which communicate with the rest of the chip via the AXI4 bus protocol. In addition to the NPU cores, the chip includes essential modules such as an RISC-V CPU, I/O interface, random-access memory (RAM), direct memory access (DMA), and other supporting components.

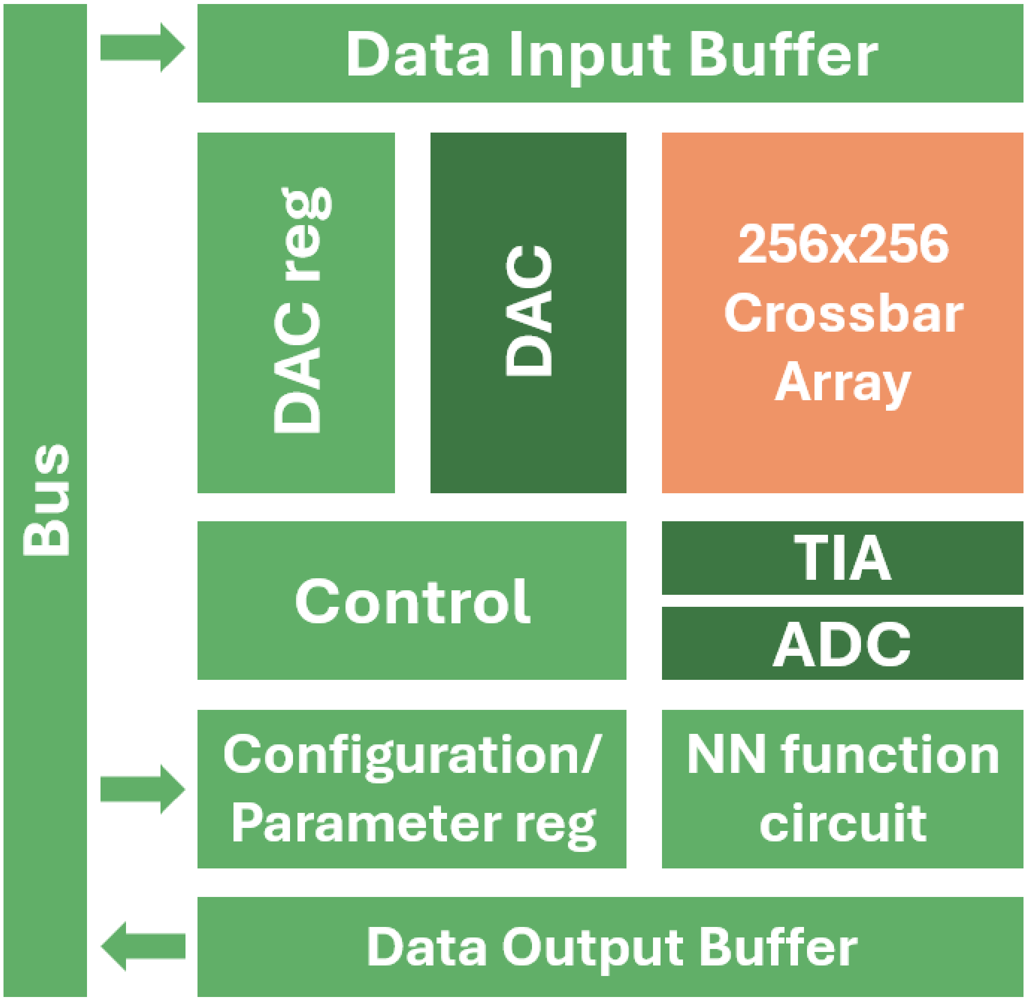

Fig. S4. Schematic of one NPU core. Each core includes a 256×256 memristor crossbar array and key modules such as digital-to-analog converters, ADC, and transimpedance amplifiers (TIA). Additionally, it incorporates peripheral circuits like buffers, registers, and neural network (NN)function circuits, ensuring seamless integration and efficient operation within the SoC.

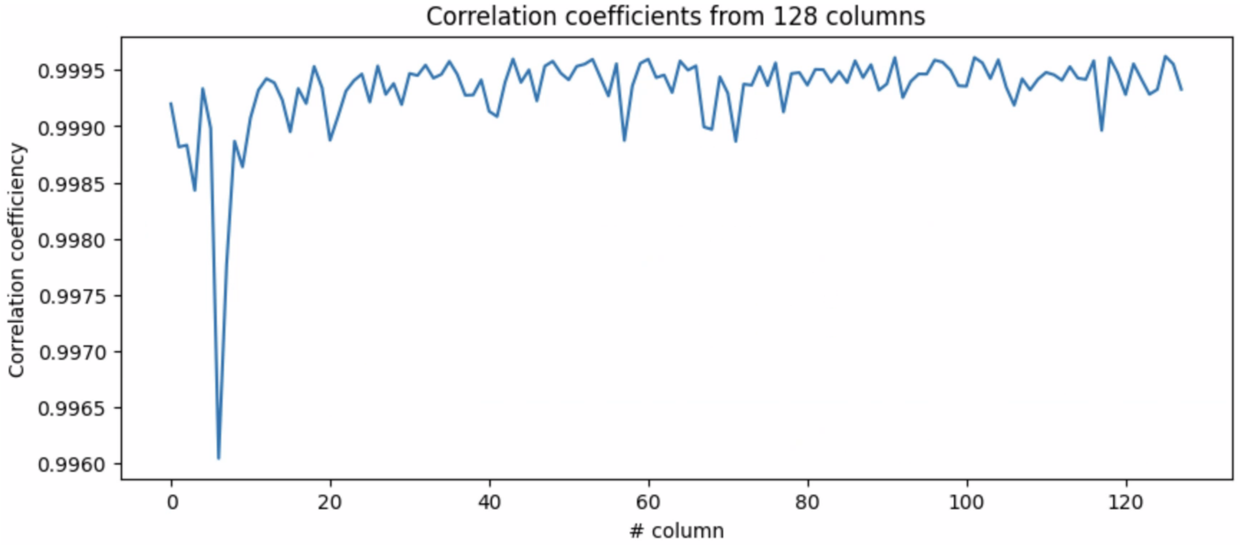

Fig. S5. Correlation coefficients across 128 columns. The plot shows the correlation coefficients calculated to 128 columns. The y-axis represents the correlation coefficient values, while the x-axis corresponds to the column index. Most columns exhibit a high correlation coefficient close to 0.995, indicating strong consistency. A slight dip in correlation is observed in certain columns, with the lowest coefficient around 0.9960. This highlights a generally robust but slightly variable correlation performance across the columns.

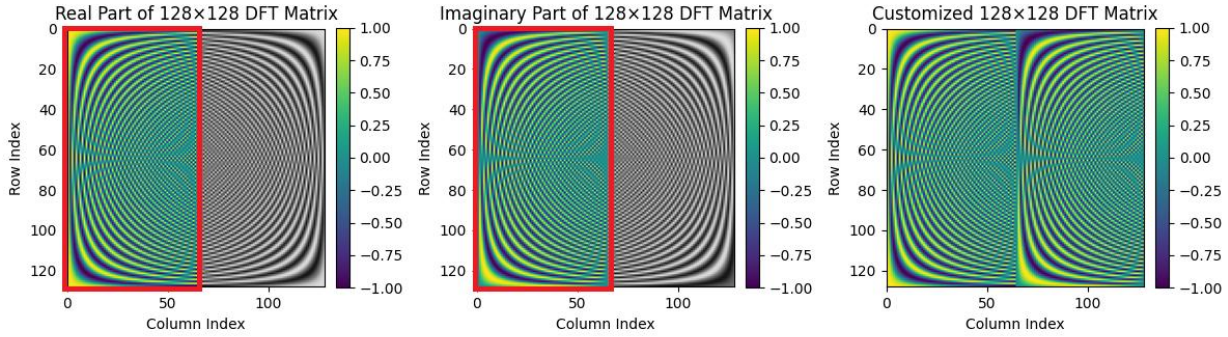

Fig. S6. Strategy for constructing the customized Discrete Fourier Transform (DFT) matrix. The first and second images depict the real and imaginary parts, respectively, of the original 128×128 DFT matrix defined by Equation (1) in the main content. The sections highlighted within the red frame contain the same information as the grayscale parts outside the frame. By combining the two colorful regions, we can form a customized DFT matrix that retains all the necessary information while reducing redundancy.

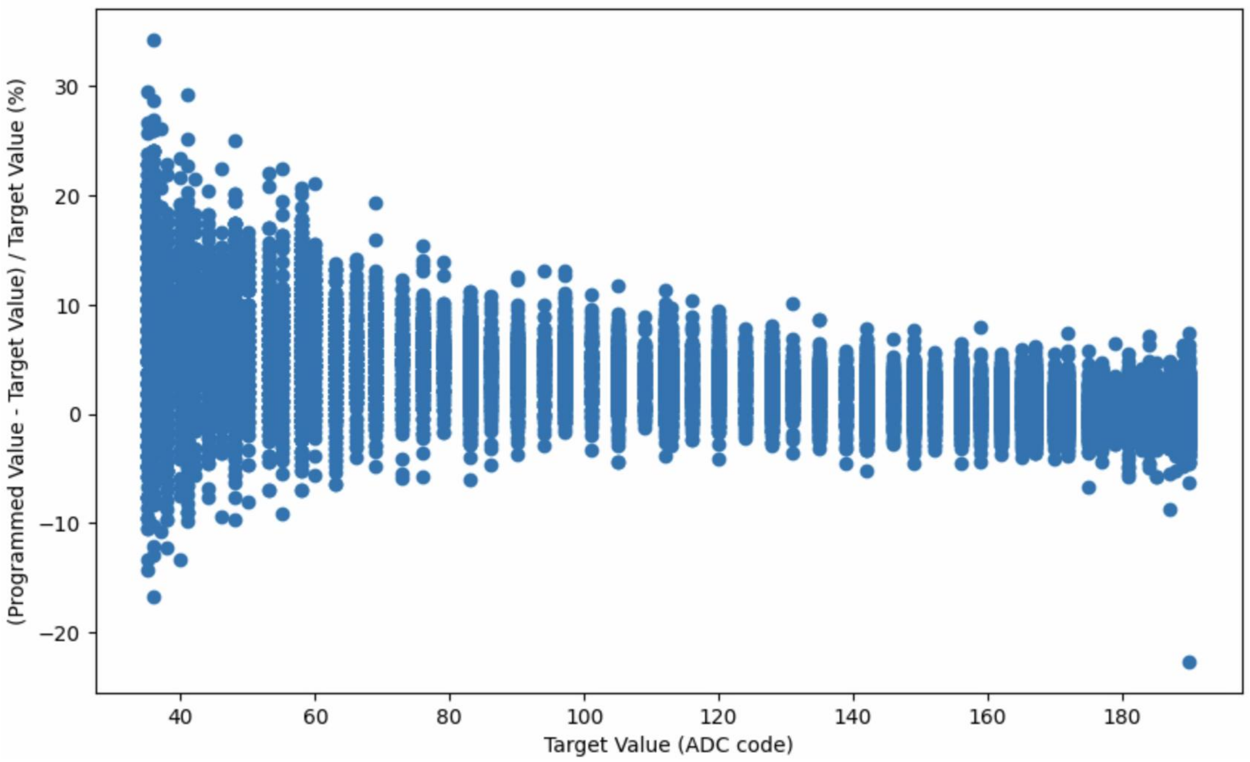

Fig. S7. Measured programming error versus target values in the DFT matrix. The programming error, defined as the difference between the programmed value and the target value divided by the target value, remains below 15% for most of the points, with a root-mean-square (RMS) error of 6.40%. This demonstrates the high precision achieved during the programming process.

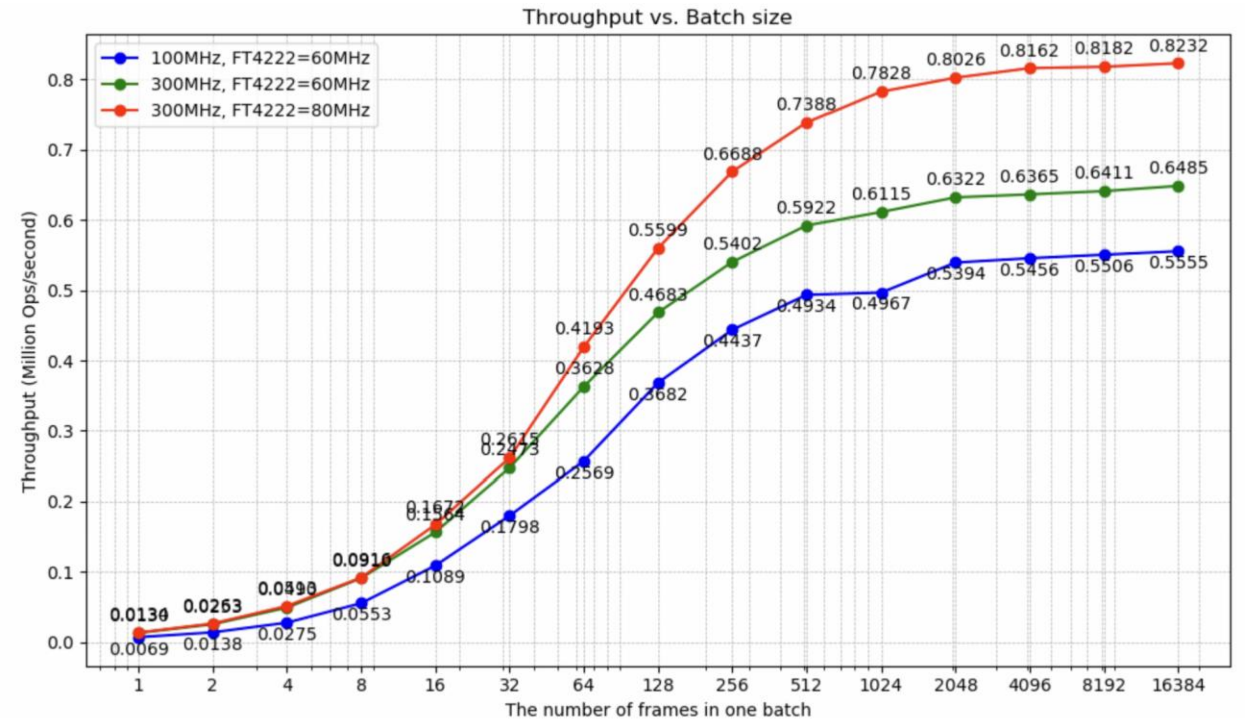

Fig. S8. Measured throughput of the SoC. Throughput is defined as the number of operations per second, where an operation corresponds to a single multiplication. For instance, a VMM operation between a vector of length N and an N×N matrix involves N^2 operations. The throughput varies depending on the CPU clock frequency, SPI clock frequency, and the number of vectors processed in a single batch. The maximum measured throughput is 0.82 MOPS (million operations per second).

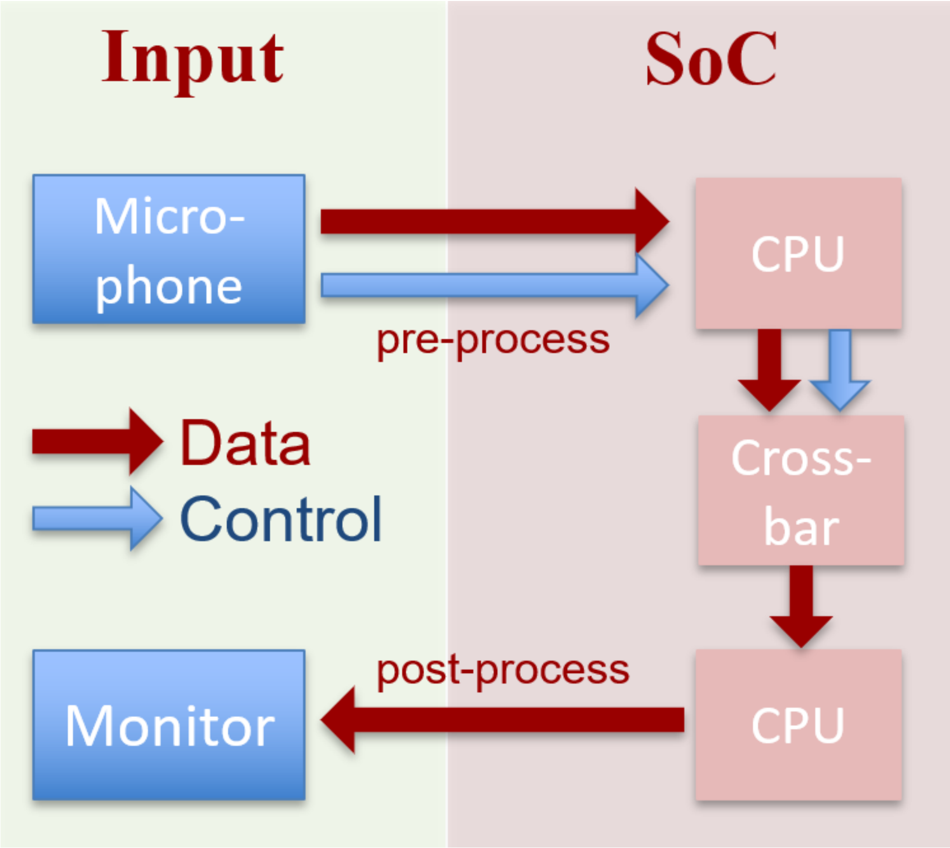

Figure S9. Structure of the real-time memristor-based spectrometer system. The system consists of a microphone for input, the SoC for signal processing, and a monitor for display. The **data flow** begins with the microphone capturing audio signals, which are pre-processed and sent to SoC. Within SoC, the CPU coordinates operations with the crossbar array to perform computations. After processing, the results are post-processed and sent back to the monitor for display.

Supplementary Note 3: Four-layer convolutional neural network (CNN) using the TetraMem Instinct 1.4 software development kit (SDK).

To classify spoken digits, a four-layer CNN was designed and trained using PyTorch. The trained network was then implemented on a SoC using the TetraMem Instinct 1.4 software development kit (SDK). The network architecture, depicted in **Fig. S10**, consists of four 1D convolutional layers followed by a fully connected layer.

The AudioMNIST dataset is an open-source audio dataset consisting of 30,000 samples of human-spoken digit in English. We use 24,000 samples for training the neural network and 6,000 samples for the inference phase in which 1,000 samples are used to calibrate ADCs on the SoC.

The input consists of audio signals of length 3600. These inputs are first processed by a DFT layer, implemented as a fixed convolutional layer with weights derived from the DFT matrix. This layer transforms the input signal into a 15 × 897 feature map, effectively capturing frequency-domain features. The transformed feature map is then passed through three sequential convolutional layers. Both convolutional layers employ clipping functions in place of ReLU activations. The clipping operation constrains the output range, enabling easier quantization and improving compatibility with the unsigned 8-bit integer arithmetic used in the SoC. To enhance efficiency and reduce overfitting, each convolutional layer is followed by max-pooling for dimensionality reduction and dropout for regularization.

The output from the second convolutional layer is flattened into a 1 × 96 vector, which is then passed to a fully connected (FC) layer. The FC layer generates a 1 × 10 output vector, corresponding to the logits for classification into 10 spoken digit classes. This streamlined architecture allows the network to process input audio signals in real time.

CNN was trained using PyTorch, with dropout applied during training to improve robustness. Additionally, 5% noise was added during training to simulate potential hardware errors, further enhancing the network's resilience. The trained floating-point model achieved a testing accuracy of 96.00%. After training, the model was quantized using the SDK, achieving a testing accuracy of 95.23%. The quantized model was then deployed on the SoC via the SDK, and real hardware testing yielded a final accuracy of 94.72%, as shown in **Fig. 3D**.

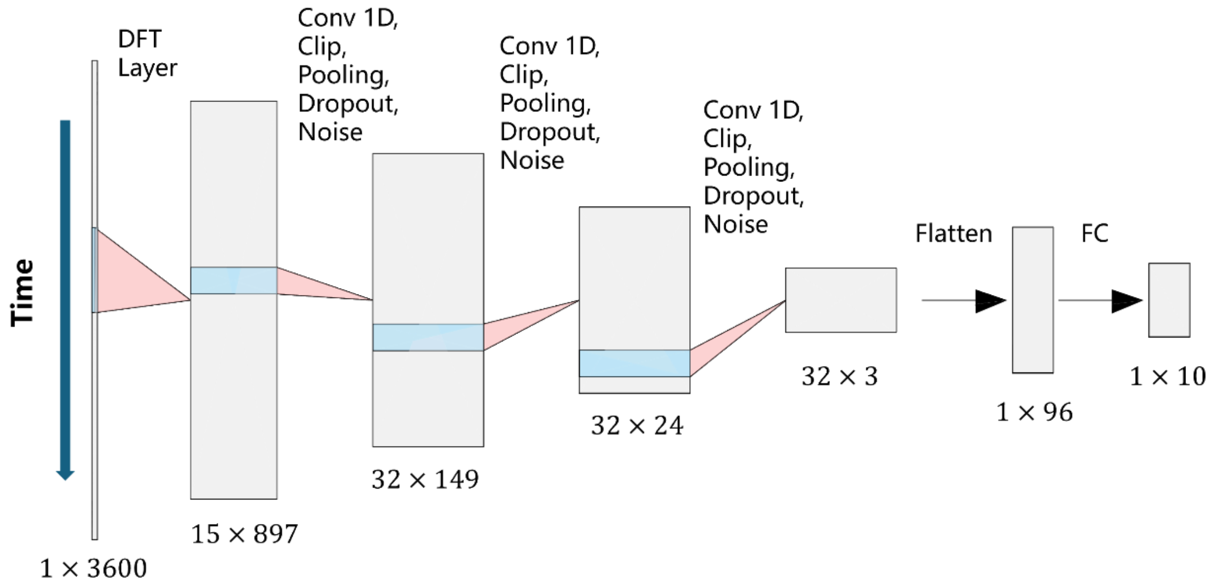

Fig. S10. Structure of the four-layer CNN. The input is a 1 × 3600 audio signal, which is processed by a DFT layer (treated as a fixed convolutional layer) to produce a 15 × 897 feature map. This is followed by three convolutional layers each incorporating clipping, max-pooling, dropout, and noise injection, progressively reducing the feature map to 16 × 10. The flattened output (1 × 96) is passed to a FC layer, producing the final output (1 × 10).

Supplementary Note 4: Error analysis on the accuracy of the edge detection task.

The discrepancy between the edge detection results obtained from software and hardware primarily arises from two sources: memristor programming errors and ADC reading errors.

For the programming error, we provide a measured distribution of the deviation between the target and programmed values in the convolution kernels, as shown in **Fig. S11**. The results indicate a mean error of 5.46 and a variance of 6.29 in ADC code. As for the ADC reading error, after applying the calibration method described in **Supplementary Note 2**, the mean error is 2.31 with a variance of 5.72 in ADC code.

To evaluate the impact of these errors on edge detection accuracy, we conducted a Python-based simulation to emulate the noise effects during convolution. First, a reference result was generated using ideal (noiseless) convolution kernels. Then, Gaussian noise was added to the kernel weights based on the measured programming error distribution, simulating the effect of memristor inaccuracy. This yielded a PSNR of 38.82 dB. Subsequently, an independent Gaussian noise component, following the ADC error distribution, was applied to the convolution output to emulate ADC reading errors. This resulted in a PSNR of 31.08 dB, which closely matches the experimentally observed value of 30.43 dB. A comparison of these results is shown in **Fig. S12**.

These simulation results confirm that the accuracy of the edge detection task is predominantly influenced by the precision of the memristor programming and ADC readout processes.

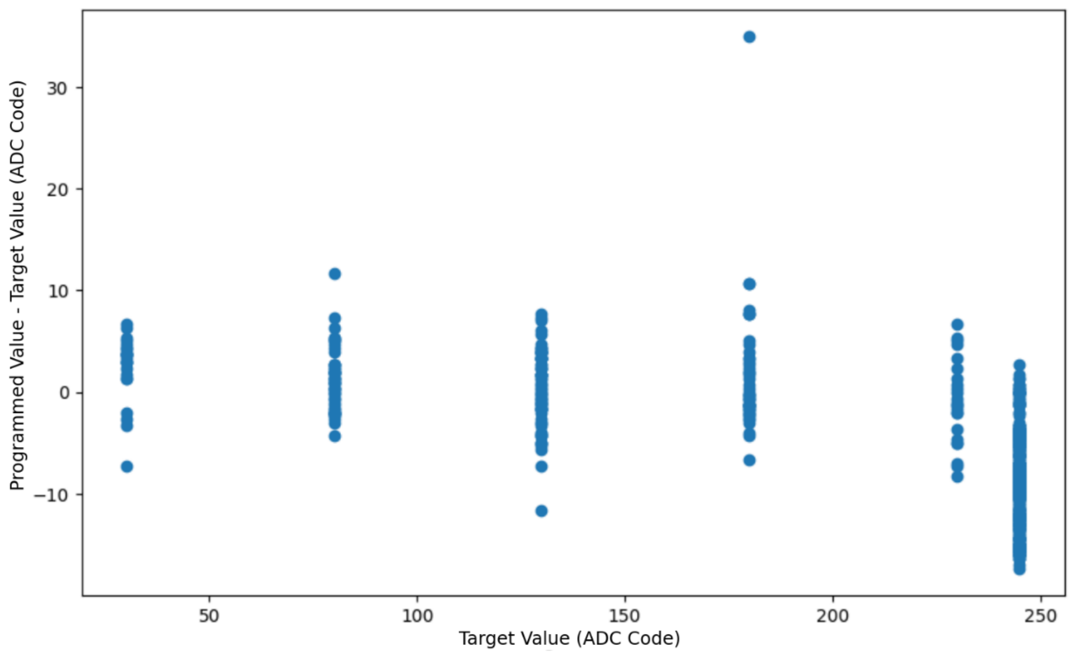

Fig. S11. Measured programming error versus target values in the convolution kernels. The programming error is defined as the difference between the programmed value and the intended target value. The measured distribution shows a mean error of 5.46 ADC codes and an RMS error of 6.29 ADC codes.

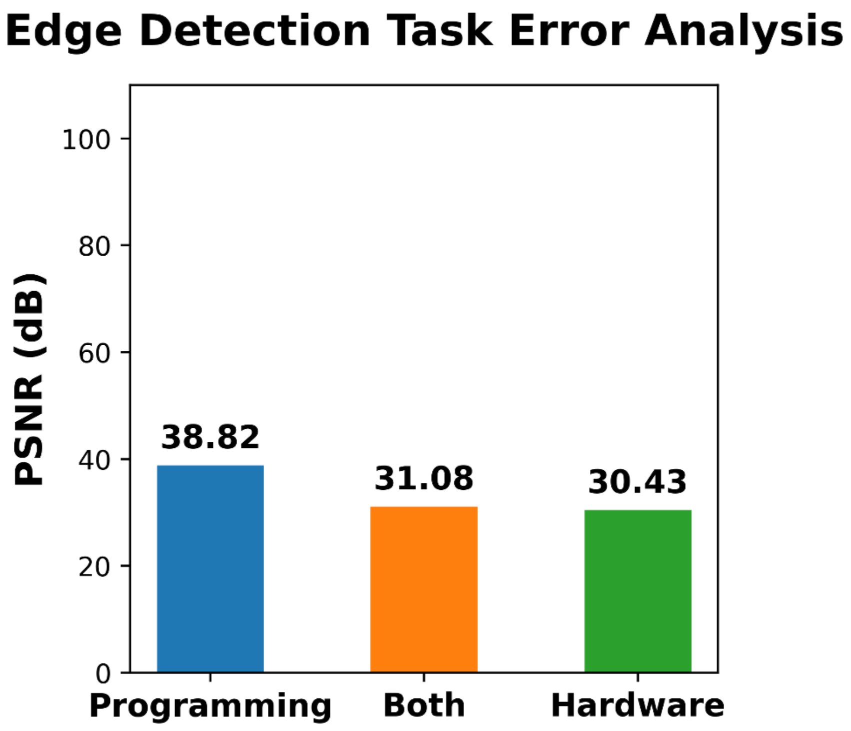

Fig. S12. Results of the edge detection error analysis. The first blue bar represents the case where only programming noise is applied to the convolution kernels, resulting in a PSNR of 38.82 dB. The second orange bar includes both programming and ADC reading noise in the simulation, yielding a PSNR of 31.08 dB. This closely matches the hardware experimental result of 30.43 dB, shown as the green bar.

Movie S1.

This video demonstrates real-time convolution implemented on our memristor-based analog SoC. The four subplots represent: (upper-left) the original frame captured by the camera, (upper-right) the frame down-sampled to 64×78, (lower-left) the edge-detected frame processed in software, and (lower-right) the edge-detected frame processed by the hardware.

Movie S2.

This is the movie for real-time spectrum calculation. The system is described in Fig. S9. The upper blue curve represents the real-time input audio signal from the microphone. The lower red and green curves are the spectrum of the input audio signal calculated by hardware and software, respectively. The sample rate of the input audio signal is 6400 Hz, which results in the Nyquist frequency of 3200 Hz. The time-domain signal is scaled to [-255, 255], while the result of the Fourier transform is scaled to [0, 255].

Movie S3.

This video shows how we do the real-time spoken digit recognition. The real-time waveform is on the left part of the computer screen, while real-time classification result is on the right part of the screen. The author directly speaks to the microphone, which transfers the audio data to PC and then to the SoC chip for calculation.
